# Safety of Anticoagulant Treatment in Patients With Splanchnic Vein Thrombosis and History of Portal Hypertension–Related Bleeding

**DOI:** 10.1111/liv.70114

**Published:** 2025-04-29

**Authors:** Rosa Talerico, Simona Pellegrino, Aurélie Plessier, Francesca Romana Ponziani, Angelo Porfidia, Francesco Landi, Antonio Gasbarrini, Roberto Pola, Francesco Santopaolo

**Affiliations:** ^1^ Department of Aging, Orthopedic, and Rheumatologic Sciences Fondazione Policlinico Universitario A. Gemelli IRCCS, Università Cattolica del Sacro Cuore Rome Italy; ^2^ Université de Paris, AP‐HP, C, DMU DIGEST, Centre de Référence Des Maladies Vasculaires du Foie, FILFOIE, ERN RARE‐LIVE. Centre de Recherche Sur L'inflammation, Inserm Paris France; ^3^ Department of Medical and Surgical Sciences Fondazione Policlinico Universitario A. Gemelli IRCCS, Università Cattolica del Sacro Cuore Rome Italy

**Keywords:** bleeding history, portal hypertension‐related bleeding, splanchnic vein thrombosis

## Abstract

Splanchnic vein thrombosis (SVT) can be associated with liver cirrhosis or prothrombotic conditions, including myeloproliferative disorders, intra‐abdominal inflammation, solid cancers or surgery. While anticoagulation therapy improves outcomes in noncirrhotic patients and reduces all‐cause mortality in cirrhotic populations, its safety in patients with a history of portal hypertension (PH)‐related bleeding is less clear. This systematic review examines the impact of anticoagulant therapy on rebleeding risk in SVT patients with a history of PH‐related bleeding. A systematic review and meta‐analysis were conducted according to PRISMA guidelines. A comprehensive search of PubMed, Web of Science and Scopus was performed for studies published up to September 2024. Studies were included if they compared SVT patients with a history of PH‐related bleeding receiving anticoagulant therapy versus those not receiving anticoagulants. The primary outcome was the cumulative incidence of PH‐related rebleedings. Of 2853 identified studies, five (186 participants) met the inclusion criteria: two randomised controlled trials (RCTs) and three observational studies. The cumulative incidence of PH‐related rebleeding was significantly lower in the anticoagulant group at 17.10% [95% CI 17.02, 17.19] compared to the control group at 40.00% [95% CI 39.90, 40.09]. The overall odds ratio (OR) from observational studies was 0.15 [95% CI 0.04, 0.52], indicating a reduced bleeding risk, while the OR from RCTs was 0.84 [95% CI 0.31, 2.32], showing a nonsignificant trend. Anticoagulant therapy may reduce rebleeding risk in SVT patients with a history of PH‐related bleeding, but further high‐quality studies are needed.

**Summary**

- Patients with splanchnic vein thrombosis (SVT) are at high risk for thrombotic and bleeding events due to portal hypertension (PH).
- This systematic review evaluates the impact of anticoagulant therapy on rebleeding risk in patients with SVT and a history of PH‐related bleeding.
- Results suggest that anticoagulant therapy may reduce the incidence of PH‐related rebleeding compared to no anticoagulation.
- The limited data and quality of evidence hinder the ability to draw definitive conclusions, underscoring the need for further research to guide clinical management of SVT and PH‐related bleeding.

**Abbreviations**
CIs: confidence intervals
CRNM: clinically relevant nonmajor
DOACs: direct oral anticoagulants
EBL: endoscopic band ligation
EVL: endoscopic variceal ligation
ISTH: International Society on Thrombosis and Haemostasis
NOS: Newcastle–Ottawa scale
NSBBs: nonselective beta‐blockers
OR: odds ratio
PH: portal hypertension
PRISMA: Preferred Reporting Instructions for Systematic Reviews and Meta‐analysis
PVT: portal vein thrombosis
RCTs: randomised controlled trials
SVT: splanchnic vein thrombosis
VKAs: vitamin K antagonists

## 1 Background

Splanchnic vein thrombosis (SVT) affects the portal vein, the mesenteric veins, the splenic vein or, in the rarest case, the suprahepatic veins (Budd–Chiari syndrome). The heterogeneity of the population with SVT relies first on the presence or absence of liver cirrhosis, and then on other underlying conditions such as solid cancer, myeloproliferative neoplasms, intra‐abdominal inflammatory conditions and surgery [1, 2]. Anticoagulant therapy has largely improved the outcomes in patients with SVT without cirrhosis and represents the mainstay of treatment [3, 4]. Two recent meta‐analyses [4, 5] assessed that anticoagulation reduces all‐cause mortality in patients with cirrhosis and portal vein thrombosis (PVT). Improving survival does not seem to be associated with recanalisation. As expected, in both meta‐analyses, nonportal hypertension (PH)‐related bleeding was more frequent with anticoagulation. To date, significant variability in the treatment of SVT persists, largely due to the diverse underlying clinical conditions and to the bleeding risk, especially related to PH. Although the risk of bleeding in patients with SVT undergoing anticoagulant therapy—particularly in those with cirrhosis—is significant, evidence suggests that bleeding is primarily related to the severity of PH and is not directly dependent on anticoagulant treatment. However, most studies have been conducted on highly selected, often small populations, or have employed a retrospective design. The primary objective of this systematic review is to assess whether anticoagulant therapy affects the likelihood of rebleeding in patients with SVT and a history of bleeding associated with PH, compared to those not receiving anticoagulant therapy. The hypothesis is that anticoagulant therapy, by promoting vessel recanalisation and preventing further thrombotic events, may reduce portal pressure and thereby lower the risk of rebleeding, particularly in cases of gastrointestinal bleeding.

## 2 Methods

This systematic review and meta‐analysis is reported according to the 2020 Preferred Reporting Item for Systematic Reviews and Meta‐Analyses (PRISMA) guidelines [6] to ensure transparency and reproducibility in the review process (Table S2) and was prospectively designed and registered on the PROSPERO international database (CRD42024589644, 8 October 2024).

### 2.1 Study Search

A comprehensive literature search of studies reporting safety data of anticoagulant therapy for the treatment of splanchnic vein thrombosis has been performed using the major electronic databases (PubMed, Web of Science, Scopus), without any language restriction (Table S3). Articles until 1 September 2024 were included.

Inclusion criteria encompassed studies that compared adults, cirrhotic and noncirrhotic, diagnosed with SVT regardless of onset time, with a documented history of PH‐related bleeding (either prior to or at the time of the SVT index event), who received anticoagulant therapy (e.g., unfractionated heparin, low molecular weight heparin, fondaparinux, vitamin K antagonists (VKAs) or direct oral anticoagulants (DOACs)) for the treatment of SVT to those who did not receive anticoagulant medications. Both observational studies and randomised controlled trials were considered. Studies that met at least one of the following exclusion criteria were excluded: (1) guidelines, expert position or consensus; case reports, letters, comments, reviews and meta‐analyses; animal studies; (2) studies that did not provide any data on rebleeding outcomes or bleeding history; (3) single‐arm studies that solely focused on anticoagulant treatment. The primary outcome was the incidence of PH‐related rebleeding. In determining cumulative incidence, all bleedings related to PH were considered, in the absence of a univocal clinical definition of PH‐related bleeding.

The secondary outcomes were major [7] and clinically relevant nonmajor (CRNM) [8] bleeding unrelated to PH according to the International Society on Thrombosis and Haemostasis (ISTH) definition and overall mortality.

Two independent reviewers (R.T. and S.P.) screened the titles and abstracts for eligibility, followed by full‐text reviews of potentially relevant articles. Discrepancies between reviewers were resolved through discussion and consultation with a third reviewer (F.S.). Selection results were reported according to the PRISMA flowchart, and the software used to record decisions was Rayyan.

For the included studies, data extraction was performed using a standardised form, capturing author, publication year, study period, study design, number of included patients and patient characteristics, details of the previous bleeding event, type of anticoagulant treatment (oral anticoagulant therapy, parenteral or both), details regarding splanchnic vein thrombosis, follow‐up duration and outcomes. We also contacted the authors of studies that featured comparative analyses of the relevant populations but did not clearly express data on rebleeding associated with PH. We have only received a response from the author of one RCT, the results of which are included in the present analysis [9].

### 2.2 Statistical Analysis and Risk‐of‐Bias Assessment

The primary outcome was assessed by calculating the cumulative incidence of bleeding related to PH for both anticoagulant and nonanticoagulant groups. This was achieved by dividing the number of cases experiencing bleeding complications by the total number of reported patients with a history of PH bleeding in both groups. For the secondary outcomes, the cumulative incidence of events was calculated in a similar way, by dividing the number of cases by the total number of reported patients.

For each study, we summarised the intervention effects by calculating odds ratios (ORs) and their corresponding 95% confidence intervals (CIs) using a random‐effects model. The evaluation of statistical heterogeneity was performed using the I^2^ statistic, with a Cochran Q test *p*‐value of less than 0.05 considered significant for heterogeneity. The I^2^ values were interpreted as follows: 0%–25% indicated insignificant heterogeneity, 26%–50% indicated low heterogeneity, 51%–75% indicated moderate heterogeneity and over 75% indicated high heterogeneity.

Additionally, publication bias was evaluated using funnel plots for bleedings related to PH of odds ratios versus standard error, and Egger's test was conducted to assess the asymmetry of the funnel plot.

Statistical analysis was conducted using RevMan version 7.12.0 and RStudio 2024.09.0.

Quality assessment of the included studies was conducted using the Newcastle–Ottawa scale (NOS) for observational studies and the Cochrane collaboration's risk of bias tool (RoB 2) for randomised controlled trials.

## 3 Results

### 3.1 Study Selection

**FIGURE 1:**
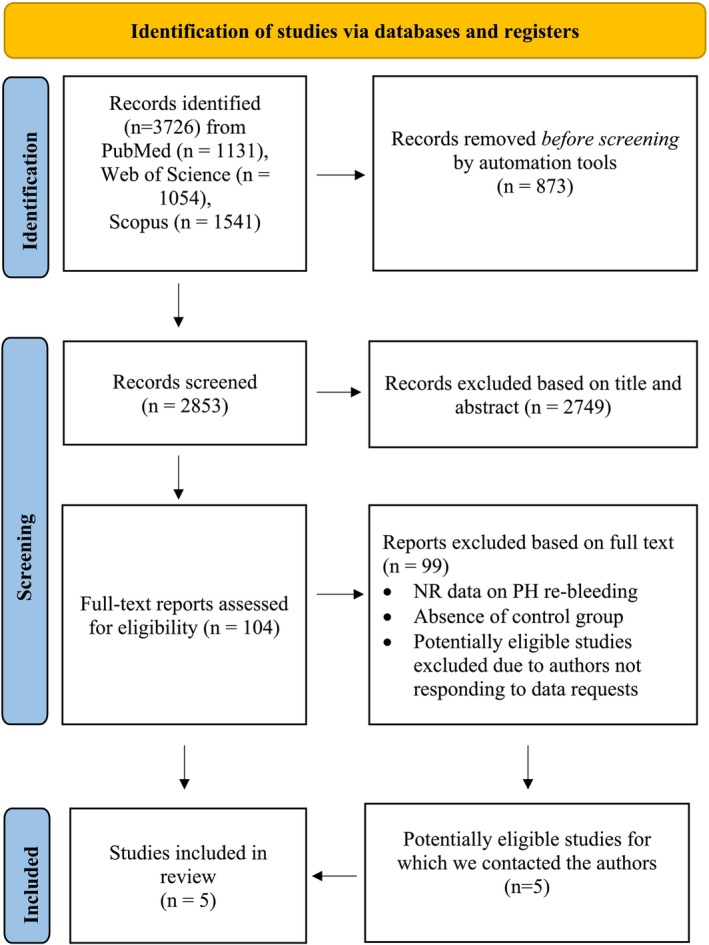
Prisma flowchart that represents the step‐by‐step process to analyse literature for the meta‐analysis. Abbreviations: NR (not reported).

Figure 1 is a visual representation of the step‐by‐step process undertaken to retrieve relevant literature for this meta‐analysis. In our comprehensive search, a total of 2853 records were initially identified as potentially relevant to the meta‐analysis. After reviewing the titles and abstracts, we excluded 2749 articles that did not align with our research objectives for the reasons previously outlined. In the second phase, we carefully examined the full texts of the remaining 104 studies to assess their suitability based on predetermined criteria. Ultimately, five studies (*n* = 186 participants) were included in the analysis. Among these, three studies (*n* = 93 participants) were nonrandomised [10, 11, 12] and two studies (*n* = 93 participants) were randomised controlled trials [9, 13]. Of the five studies included, two examined patients with liver cirrhosis, two focused on noncirrhotic patients and one analysed a mixed population that included both cirrhotic and noncirrhotic individuals. Regarding the type of anticoagulant therapy administered, three studies employed VKAs [10, 11, 13], while only one employed DOACs (i.e., Rivaroxaban) [9]. One article did not specify the type of anticoagulant therapy administered [12]. The baseline characteristics and outcomes of the studies included in the analysis are reported in Table 1.

**TABLE 1:** Baseline characteristics and outcomes of the included studies in this meta‐analysis.

Author, year	Type of study	Enrolment period	Follow‐up, mo^a^	Aetiology	Type of SVT	Sample size, *n* (AC/C)	Sample size with history of PH‐ bleeding, *n* (AC/C)	Type of AC therapy	PH rebleeding, *n* (AC/C)	Overall MB, *n* (AC/C)	Mortality, *n* (AC/C)
Orr et al., 2007 [10]	R NRS, single centre	January 1973–June 2015	42	Both	MVT	60 (18/42)	32 (5/27)	VKAs	16 (0/16)	NR	NR
Zhou et al., 2023 [11]	R NRS, single centre	January 2015–December 2019	51 (AC) 44 (C)	Cirrhotic	PVT	84 (46/38)	34 (18/16)	VKAs	11 (3/8)	NR	NR
Spaander et al., 2008 [12]	R NRS, single centre	January 1982–October 2005	60	Noncirrhotic	PVT	27 (5/22)	27 (5/22)	NR	10 (0/10)	NR	NR
Gao et al., 2022	RCT, multicentre	NR	6	Cirrhotic	PVT	86 (43/43)	86 (43/43)	LMWH (nadroparin) + VKAs	17 (8/9)	0	0
Plessier et al., 2022 [9]	RCT, multicentre	September 2015–January 2020	30.3	Noncirrhotic	PVT	111 (55/56)	7 (5/2)	DOACs (Rivaroxaban)	3 (2/1)	NR	0

Abbreviations: AC, anticoagulant group; C, control group; DOACs, direct oral anticoagulants; LMWH, low molecular weight heparin; MB, major bleeding; mo, months; MVT, mesenteric venous thrombosis; NR, not reported; NRS, nonrandomised study; PH, portal hypertension; PVT, portal vein thrombosis; R, retrospective observational study; RCT, randomised controlled trial; VKAs, vitamin K antagonist.

^a^ Referred to entire sample size.

The quality of observational studies and Rob2 results for the RCTs are reported in the Supporting Information (Table S1 and Figure S1 respectively).

### 3.2 Primary Outcome

The primary outcome was the incidence of PH‐related rebleeding, primarily indicated by bleeding from oesophageal varices in most of the included studies [10, 11, 13]. In addition, one study reported bleeding from gastric varices [12]. The events of each study are listed in Table 1.

The cumulative incidence of PH‐related rebleeding was lower in the group of patients who were on anticoagulant therapy, at 17.10% [95% CI 17.02, 17.19] compared to 40.00% [95% CI 39.90, 40.09] in the control group (patients not receiving anticoagulants). When combining the results according to study type, we found that the cumulative incidence of rebleeding in observational studies was 10.71% [95% CI 0.0, 22.05] in the anticoagulated group versus 52.31% [95% CI 40.36, 64.27] in the control group, whereas in RCTs it was 20.83% [95% CI 9.60, 32.06] in the group receiving anticoagulant medication versus 22.22% [95% CI 9.30, 35.14] in the control group.

**FIGURE 2:**
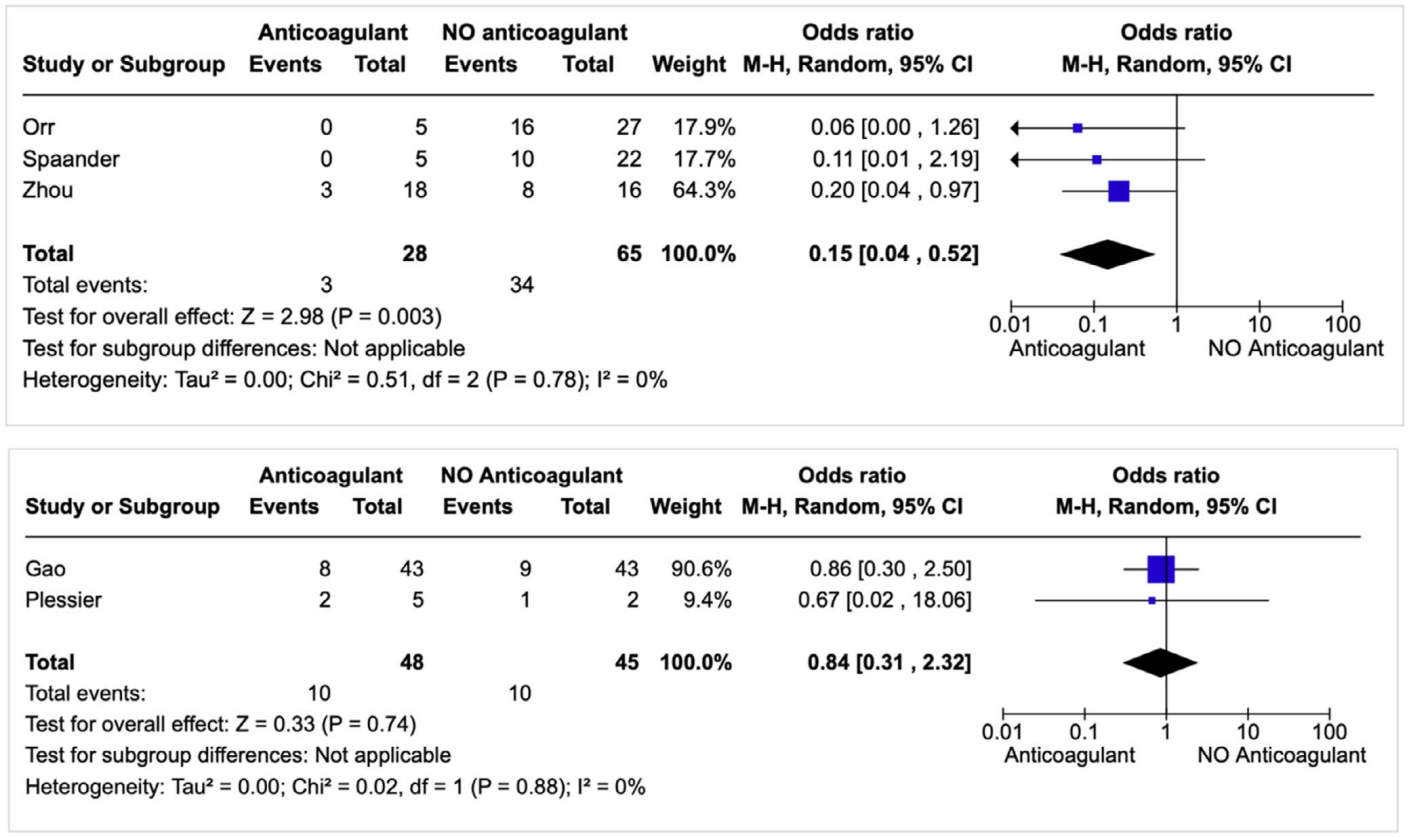
PH rebleeding between AC group versus control group in patients with SVT and a history of PH‐related bleeding (random‐effects model) in the NRSs (above) and RCTs (below). Abbreviations: AC (anticoagulant group), C (control group), CI (confidence interval), NRSs (Nonrandomised Studies), PH (portal hypertension), RCTs (randomised controlled trials), SVT (splanchnic vein thrombosis).

When we pooled data from various observational studies, we calculated an overall OR of 0.15 [95% CI 0.04, 0.52], indicating that the risk of bleeding was significantly lower for the anticoagulant group compared to the control group (Figure 2). The OR from the RCTs was 0.84 [95% CI 0.31, 2.32], indicating a nonsignificant trend toward a reduced risk of bleeding during anticoagulant therapy (Figure 2).

Additionally, no significant heterogeneity was observed in the results of both the observational clinical studies and RCTs, as indicated by an I^2^ of 0% and *p* values of 0.78 and 0.88 respectively (Figure 2). The funnel plot analysis indicated a slight asymmetry, which is likely due to heterogeneity among the studies; however, there is no clear evidence of publication bias (*p* = 0.1977) (Figure S2).

Finally, due to the small sample size, no subgroup analysis was conducted between the cirrhotic and noncirrhotic populations.

### 3.3 Management of Portal Hypertension

In the RCT by Gao et al. [13], all patients in both groups were managed with endoscopic band ligation (EBL) every 28 days until variceal eradication was achieved, alongside treatment with nonselective beta‐blockers (NSBBs) for secondary prophylaxis against variceal rebleeding. In contrast, the study by Spandeer et al. [12] also employed EBL as treatment for all patients, but only 10 patients (group assignment not specified) received NSBB therapy. The approach to secondary prophylaxis in the other studies remains unclear, as it is not explicitly stated whether all the patients in the subpopulation of interest were treated with NSBBs or other measures to prevent variceal rebleeding.

### 3.4 Secondary Outcomes

With respect to the secondary outcomes, the observational studies [10, 11, 12] included in this review did not provide specific data on major and CRNM bleeding unrelated to PH, or mortality. As a result, it was not feasible to conduct such an analysis.

In the population of the randomised clinical trial by Gao et al. [13], no episodes of major bleeding, CRNM bleeding or death were recorded in either group during the observation period (Table 1). No deaths were reported in the study by Plessier et al. [9], but it is not clearly stated whether major or CRNM bleeding unrelated to PH events occurred in the population of interest.

## 4 Discussion

Anticoagulant therapy is the cornerstone of treatment of SVT and has been shown to improve outcomes in patients with SVT without cirrhosis [14] and to reduce all‐cause mortality in those with cirrhosis and portal vein thrombosis [3]. However, it is well known that patients diagnosed with thrombosis of the splanchnic veins, regardless of aetiology, are at high risk for gastrointestinal bleeding related to the development or worsening of PH [15]. In noncirrhotic patients with PVT, the likelihood of developing varices is 22% within 3 years. Furthermore, the risk of bleeding due to PH in patients receiving primary prophylaxis is 20% at 3 years [16], similar to the rates observed in patients with cirrhosis [16, 17, 18, 19]. This poses a considerable challenge to clinicians.

The results of our meta‐analysis indicate that the cumulative incidence of PH‐related rebleeding in patients with SVT is lower among those receiving anticoagulant therapy compared to untreated individuals. This finding may be linked to the impairment of portal haemodynamics caused by thrombosis of the splanchnic district. Specifically, SVT could worsen PH, lead to the enlargement of oesophagogastric varices and increase the risk of variceal rupture. Conversely, the recanalisation of the affected vessel, achieved through anticoagulation, may alleviate PH, thereby explaining the reduced incidence of rebleeding observed in treated patients [20]. Although the findings were more evident in observational studies, which may have confounding limitations compared to RCTs, they suggest that anticoagulant therapy does not significantly elevate the bleeding risk associated with PH. Additionally, a significant concern lies in the difference in sample size between the two included RCTs, which may have substantially affected the analysis. Furthermore, the selective criteria that researchers have employed over the years when determining candidates for anticoagulant therapy in the observational analysed studies may also influence these results. Therefore, while anticoagulation may be regarded as a safe option for properly managed patients, it is crucial to interpret this conclusion with caution.

The trend observed in our study in favour of the use of anticoagulant therapy in patients with SVT and a history of PH bleeding is consistent with previous evidence in the literature. A meta‐analysis by Loffredo et al. [21] showed that, despite no significant difference in major and minor bleeding rates between anticoagulant‐treated and untreated patients with cirrhosis and PVT (11.00% for both groups), the rate of variceal bleeding was significantly lower in the anticoagulant group (2.00%) compared to the untreated group (12.00%) (OR 0.23; *p* value = 0.04). A similar result emerged from a subsequent meta‐analysis by Gao et al., which included a larger number of studies and documented a significantly lower incidence of PH‐related bleeding in patients treated with anticoagulants for PVT, compared to untreated ones (OR = 0.21; *p* value < 0.001) [22]. The same finding was reported by Hall et al. [23] in a population of noncirrhotic PVT, who showed that anticoagulant therapy was protective against variceal bleeding. Indeed, this evidence supports the hypothesis that bleeding would be independent of anticoagulant treatment and mainly related to the severity of PH. This validates the view that anticoagulation in SVT patients and clinically significant PH with a history of bleeding could be as safe as in patients without such a history, provided that adequate secondary prophylaxis of variceal bleeding is performed before starting anticoagulation [24, 25]. The Baveno VII consensus [24] offers guidelines for managing bleeding from gastroesophageal varices; however, it does not specifically address patients receiving anticoagulant therapy. There is insufficient data regarding the timing of resumption of anticoagulant therapy after an acute bleeding episode and the follow‐up of endoscopic procedures during anticoagulant treatment. This highlights an unmet clinical need, underscoring the necessity for further studies to provide clear recommendations for effectively managing these patients. On this note, the 2020 Practice Guidance of the American Association for the Study of Liver Diseases (AASLD) [26] recommends early anticoagulation in patients with PVT, even before complete endoscopic variceal eradication is achieved. In fact, recent studies, including one by Guillaume et al., have shown that anticoagulation does not elevate the risk of postendoscopic variceal ligation (EVL) upper gastrointestinal bleeding in noncirrhotic PVT patients compared to untreated individuals [27]. Additionally, a study by Dell'Era et al. indicated that while PVT does not seem to impact the effectiveness of EVL in either cirrhotic or noncirrhotic patients, it does prolong the time required for variceal eradication [28].

The key strength of this study lies in its evaluation of how history of prior bleeding related to PH can impact the risk of subsequent PH‐related bleeding in patients with SVT, both with and without anticoagulant therapy. Given the limited available data, this serves as an incentive for future studies to assess the history of PH‐related bleeding in SVTs and to provide more precise recommendations for managing PH in patients on anticoagulant therapy.

This study has several limitations; first, it includes a small number of studies, mostly observational in nature and conducted on a limited sample of patients. Only one study is a RCT specifically designed to assess rebleeding as the primary outcome in a cohort of patients with SVT and a history of variceal bleeding at baseline. The remaining studies provide data on rebleeding related to anticoagulant therapy in subgroups of patients with a history of PH‐related bleeding, but they do not explicitly analyse rebleeding as the primary outcome. Furthermore, as previously mentioned, the second RCT considered [9] had very few eligible patients, which may further limit the applicability of the findings.

A second limitation is the poor quality of the included studies. Observational studies suffer from inherent biases, such as confounding factors and issues with data collection. These aspects may affect the accuracy and generalisability of the results. Moreover, one of the RCTs [13] included in the analysis is not free from methodological flaws and carries a high risk of bias. Another concern is the lack of clear specifications regarding PH management, which can compromise the interpretation of the results. This ambiguity introduces potential biases and limits the ability to compare the effectiveness of different interventions. Additionally, another limitation is the lack of subgroup analysis categorised by the aetiology of SVT (cirrhotic vs. noncirrhotic populations) and the site of SVT, largely due to the limited sample size.

Furthermore, due to the scarcity of data and the overall small number of patients studied, it was not feasible to perform a subgroup analysis comparing different anticoagulant regimens.

While the majority of patients in the studies received VKAs, there is a growing trend towards the use of DOACs in clinical practice for patients with SVT [29]. Consequently, data on this type of anticoagulant therapy are limited in this analysis.

## 5 Conclusion

This meta‐analysis indicates that, in patients with SVT and a history of bleeding related to PH, anticoagulant therapy may lower the rate of rebleeding related to PH. However, the quality of evidence is low and the available data are scarce. Therefore, it is not possible to draw firm conclusions on this topic. Moreover, there is insufficient evidence to recommend the use of one specific anticoagulant over another in these patients.

Further research is needed.

## Author Contributions

R.T., S.P. and F.S. designed the study protocol, performed the search, screened for eligible studies and performed data extraction. R.T. and S.P. performed all statistical analyses and drafted the manuscript. A.P. contributed to data collection. All authors interpreted the results, reviewed drafts and approved the final draft of the manuscript.

## Ethics Statement

The authors have nothing to report.

## Consent

The authors have nothing to report.

## Conflicts of Interest

The authors declare no conflicts of interest.

## Supporting information


Data S1.

## Data Availability

The data that support the findings of this study are openly available, see reference numbers for more details.
